# Foot Disorders in Nursing Standing Environments: A Scoping Review Protocol

**DOI:** 10.3390/nursrep11030055

**Published:** 2021-07-21

**Authors:** Rafael A. Bernardes, Pedro Parreira, Liliana B. Sousa, Minna Stolt, João Apóstolo, Arménio Cruz

**Affiliations:** 1 The Health Sciences Research Unit: Nursing (UICISA:E), Nursing School of Coimbra (ESEnfC), 3004-011 Coimbra, Portugal; parreira@esenfc.pt (P.P.); baptliliana@esenfc.pt (L.B.S.); apostolo@esenfc.pt (J.A.); acruz@esenfc.pt (A.C.); 2 Department of Nursing Science, University of Turku, FI-20014 Turku, Finland; minna.stolt@utu.fi

**Keywords:** foot health, foot diseases, nurses, standing, prolonged standing

## Abstract

Musculoskeletal disorders can be significantly disabling, particularly those related to work, when the underlying mechanisms and clinical variables are not well known and understood. Nurses usually remain in standing positions or walk for long periods, thus increasing the risk for the development of musculoskeletal disorders, particularly on the foot, such as plantar fasciitis or edema. This type of disorders is a major cause of sickness, absence from work, and also dropout ratios among nursing students, which contributes to the shortage of nursing professionals. This review will address foot disorders that arise from prolonged standing in nursing professionals and describe the main clinical parameters characterizing them, with exclusions for other health professions or disorders with other identified causes. English, French, Portuguese, and Spanish published studies from 1970 to the current year will be considered. The review will follow the JBI methodology, mainly though the PCC mnemonic, and the reporting guidelines for Scoping Reviews. The search will include main databases and relevant scientific repositories. Two independent reviewers will analyze the titles, abstracts, and full texts. A tool developed by the research team will aid in the data collection.

## 1. Introduction

Musculoskeletal disorders (MSDs) can be described as health problems related to the locomotor system, affecting muscles, joints, tendons, the skeleton system, the vascular system, ligaments, and nerves [1,2]. When related to a professional activity or work-related event, MSDs are named work-related musculoskeletal disorders (WMSDs) [3], which represent a major occupational health concern for the nursing profession [4,5], accounting for 60% of the reported health occupational injuries [3].

In this sense, nurses are considered to be at a high risk of WMSDs [2,4,5], namely having their feet exposed to prolonged standing and walking for long distances [6], with a high prevalence of MSDs, when compared to other professions [5,7,8].

‘Prolonged standing’ and ‘prolonged walking’ concepts can be included in a broader definition—‘standing environments’. According to Anderson, Nester, and Williams [9], ‘prolonged standing’ is defined as spending at least 5% of occupational time standing. Regarding ‘prolonged walking’, Stolt and colleagues [1] report that nurses walk an estimated distance of 4–5 miles in a 12 h shift, thus spending most of their time working time on their feet.

‘Standing environments’ characterize a potentially aggressive context in the nursing profession, particularly regarding foot health. In fact, ‘prolonged standing’ has a 1.7-fold risk for foot pain [9,10] and, according to Reed and colleagues [5], foot/ankle MSDs were the most prevalent conditions experienced by nurses in the previous 7 days, the second most prevalent MSD to impair nurses’ physical activity, and the third most prevalent MSD after lower-back and neck problems. Additionally, more than 50% of nurses reported foot/ankle MSDs in the preceding 12 month period [1,5].

Feet-related and lower-limb conditions, being the most common [11,12,13,14], contribute to the incidence, in the nursing profession, of lesions such as plantar fasciitis, muscle fatigue, varicose veins, and edema [7,14,15].

As stated by a recent narrative literature review in lower extremity MSDs in nurses [1], little is known about what types of lower-extremity problems nurses are facing during their working time and studies with an exclusive focus on feet disorders in nurses are scarce.

Apart from nurses’ personal health and quality of life, MSDs seem to be the leading cause of sickness-related absence from work worldwide [1,16,17], also reducing worker productivity [18]. As a matter of fact, in the Fifth European Working Conditions Survey [19], it is stated that exposure to physical risk factors can negatively impact workers’ health and well-being, which is one of the European main social policies and a European Union (EU) core competence. Furthermore, the demanding physical workload that leads to MSDs is usually a cause of nursing student dropout and early exit of nurses starting their careers, thus contributing to the shortage of nursing professionals [20].

In this sense, and although several studies have identified feet problems as MSDs among nurses, a preliminary search of PROSPERO, MEDLINE, the Cochrane Database of Systematic Reviews, and the JBI Evidence Synthesis has revealed that there are no conducted, current or underway, scoping or systematic reviews that clearly describe the foot disorders in nursing professionals and related clinical parameters. Moreover, there are no studies that clearly identify the causes of pain on the foot. On the other hand, podiatric evaluations are poor, which limit a more detailed knowledge of the phenomenon under study.

Thus, in order to develop future interventions to address this issue, it is important to map the foot disorders in nursing standing environments and their main clinical parameters, which are the main objective of this scoping review.

## 2. Review Question(s)

The review questions are ‘What foot disorders do nurses who work in standing environments have?’ and ‘Which are the main clinical parameters that characterize those foot disorders?’.

## 3. Materials and Methods

The proposed scoping review will be conducted in accordance with the JBI methodology for scoping reviews [21,22], considering the PCC mnemonic, where P stands for ‘participants’, C for ‘concept’, and C for ‘context’.

Regarding participants, this review will consider studies that include all nursing professionals who are exposed to standing environments in acute care contexts, namely, hospital units. The review will exclude other health professionals, or those who usually work in a stationary environment for most of the time (e.g., clinical appointments, primary care).

The concept in study refers to ‘foot disorders’. It is widely considered that the foot is one of the most dynamic structures in the human body, acting in concert with the rest of the body during standing and movement [23]. According to Hagedorn and colleagues [24], foot disorders are related to foot posture and foot function and appear in the presence of an imbalanced event between the various internal structures, including the ankle, causing a structural lesion or affecting tendons and ligaments, also involving pain. Thus, in this review, we will address disorders of the foot/ankle as a whole, including pain as an isolated disorder, as it is the most frequent reported symptom.

As for the context, this review will consider studies that address disorders that occur within standing environments, as defined earlier in this paper. Further contextual descriptions and elements that might enrich the definition of ‘standing environments’ will also be reviewed and reported by the authors.

### 3.1. Types of Sources and Search Strategy

This scoping review will consider quantitative, qualitative, and mixed methods study designs for inclusion. In addition, systematic reviews and narrative review papers will be considered for inclusion in the proposed scoping review.

Quantitative studies to include are those considered to have any experimental study design (e.g., randomized controlled trials, quasi-experimental studies) and also observational studies (e.g., descriptive, cohort, cross-sectional studies).

Qualitative studies include those that are mainly focused on qualitative data, such as phenomenology and grounded theory, for example.

The search strategy will aim to locate both published and unpublished primary studies, reviews, text, and opinion papers. An initial limited searched of MEDLINE (PubMed) was undertaken to identify articles on the topic. The text words contained in the titles and abstracts of relevant articles and the index terms used to describe the articles were used to develop a full search strategy for PubMed (Table 1). The search strategy, including all identified keywords and index terms, will be adapted for each included information source. The reference lists of articles selected in the study will be screened for additional papers on the topic of interest.

Articles published in English, Portuguese, Spanish, and French will be included. Articles published from 1970 to the present will be included, as the oldest nursing theory and model for occupational health nursing dates from 1977 [25]. In this sense, we believe that the topic of nursing personnel’s health and self-care related to work was more present in the scientific community from the 1970s onward.

The databases to be searched include MEDLINE, CINAHL, Latindex, SciELO, Web of Science, Cochrane Database of Systematic Reviews, JBI Database of Systematic Reviews and Implementation Reports, and PROSPERO. Sources of unpublished studies and gray literature to be searched include Google Scholar, Open Grey, OpenDOAR, and ProQuest Dissertation and Theses.

### 3.2. Study/Source of Evidence Selection

Following the search, all identified records will be collated and uploaded into Mendeley and duplicates removed. Following a pilot test, titles and abstracts will then be screened by two independent reviewers for assessment against the inclusion criteria for the review. Potentially relevant papers will be retrieved in full and their citation details imported. The full text of selected citations will be assessed in detail against the inclusion criteria by two independent reviewers. Reasons for exclusion of full-text papers that do not meet the inclusion criteria will be recorded and reported in the scoping review. Any disagreements that arise between the reviewers at each stage of the selection process will be resolved through discussion or with a third reviewer. The results of the search will be reported in full in the final scoping review and presented in a Preferred Reporting Items for Systematic Reviewers and Meta-analyses for Scoping Reviews (PRISMA-ScR) flow diagram [26]. Possible disagreements between reviewers will be resolved through discussion or with the inclusion of a third reviewer.

As an additional methodological step, studies identified from the reference list of previous included studies will be assessed based on title and abstract.

### 3.3. Data Extraction

Data will be extracted from papers included in the scoping review by two independent reviewers using a data extraction tool developed by the reviewers. The data extracted will include specific details about the population, concept of interest, and context relevant to the review question. A draft extraction tool is provided (Table 2). The draft data extraction tool will be modified and revised as necessary during the process of extracting data from each included paper. Modifications will be detailed in the full scoping review. Any disagreements that arise between the reviewers will be resolved through discussion or with a third reviewer. Authors of papers will be contacted to request missing or additional data, where required.

### 3.4. Data Analysis and Presentation

Data will be presented in a tabular manner, taking into account the study research question. A descriptive summary will follow the results, unfolding how they answer the proposed questions.

Data will be summarized through the following information: author(s), year of publication, country of origin, purpose, population, sample size, methodology, walking hours (if applicable), standing hours (if applicable), foot disorders, and respective clinical parameters.

## 4. Contributions to the Topic

Differences in the way of defining foot disorders, combined with the multiple compound working contexts and personal traits, generate a great variability of clinical podologic prevalence rates, thus increasing complexity when interventions are needed at an occupational level.

With the conduction of this scoping review, we expect to further enhance the knowledge related to nurses’ foot disorders in standing environments, particularly defining characteristics and related phenotypic clinical parameters in this population.

Additionally, the intended description will allow a better understanding of the influence of nursing labor contexts on foot health and consequently provide guiding principles for further research and for therapeutic interventions within the scope of occupational health.

## Figures and Tables

**Table 1:** Search strategy for MEDLINE (PubMed) conducted on 29 December 2020.

Search	Query	Records Retrieved
#1	((((((nurs*[Title/Abstract]) OR (“nurse practitioner”[Title/Abstract])) OR (“nurse practitioners”[Title/Abstract])) OR (“nursing personnel”[Title/Abstract])) OR (“registered nurses”[Title/Abstract])) OR (“registered nurse”[Title/Abstract])) OR (nurse[MeSH Terms])	436,175
#2	(“foot disorders”[Title/Abstract] OR “foot health”[Title/Abstract] OR “foot diseases”[Title/Abstract] OR “foot disease”[Title/Abstract] OR “foot diseases”[MeSH Terms])	17,648
#3	(“standing”[Title/Abstract] OR “long standing”[Title/Abstract] OR “prolonged standing”[Title/Abstract] OR “prolonged walking”[Title/Abstract] OR “standing position”[Title/Abstract] OR “standing positions”[Title/Abstract])	75,039
#4	#1 OR #2	453,583
#5	#2 OR #3	92,458
#6	#1 OR #3	509,912
#7	#4 AND #5 AND #6	1761
Limited to Portuguese, English, French, and Spanish results.		
Limited to studies from 1970 until the present.		

**Note.** A similar strategy will be used for the remaining databases.

**Table 2:** Data extraction instrument.

Study Details	Retrieved Data
Author(s)	
Year of Publication	
Country of Origin	
Purpose	
Population	
Sample Size	
Gender	
Footwear Specifications (If Applicable)	
Methodology	
Walking Hours (If Applicable)	
Standing Hours (If Applicable)	
Foot Disorders and Respective Clinical Parameters	

## Data Availability

The data presented in this study are all available within this manuscript.
